# Examining pathogenic *Vibrio* abundance and environmental controls across macroalgae, sediment, and oysters

**DOI:** 10.1128/aem.02580-25

**Published:** 2026-04-17

**Authors:** Alexandra H. Geisser, Abigail K. Scro, Roxanna Smolowitz, Robinson W. Fulweiler

**Affiliations:** 1 Department of Biology, Boston University 1846, Boston, Massachusetts, USA; 2 Aquatic Diagnostic Laboratory, Center for Economic and Environmental Development, Roger Williams University 1992, Bristol, Rhode Island, USA; 3 Department of Earth & Environment, Boston University 1846, Boston, Massachusetts, USA; Norwegian University of Life Sciences, Ås, Norway

**Keywords:** *Vibrio vulnificus*, *Vibrio parahaemolyticus*, sediment, oyster, pathogen, macroalgae, *Vibrio*, estuary

## Abstract

Climate change is accelerating the geographic spread and abundance of pathogenic *Vibrio* species in coastal waters, posing growing threats to ecosystems and human health. We investigated the abundance and pathogenic gene presence of *V. vulnificus* and *V. parahaemolyticus* across three common estuarine substrates (macroalgae, sediment, and oysters) in a temperate estuary. Macroalgae consistently harbored the highest *Vibrio* counts in conjunction with elevated pathogenic gene frequencies, while oysters contained the lowest. Environmental conditions related to *Vibrio* abundance were substrate and *Vibrio* species specific. Temperature and salinity were most closely related to pathogenic *Vibrio* abundance in oysters. In contrast, pathogenic *Vibrio* abundance on macroalgae and within surface sediments was most closely related to water column nutrient concentrations, chlorophyll-a, and total suspended solids (TSS). Recognizing the different, substrate-specific environmental conditions related to pathogenic *Vibrio* abundance is critical for predicting risks, improving water quality models, and public health in a changing climate.

**IMPORTANCE:**

Pathogenic *Vibrio* bacteria are ubiquitous in marine and estuarine environments and can cause severe human illness, yet ecological understanding and risk assessments often focus primarily on oysters in *Vibrio* monitoring due to their relevance in seafood safety. However, oysters represent only one of the many environmental niches of *Vibrio* in coastal ecosystems. This study revealed that within this coastal system, macroalgae harbored higher *Vibrio* concentrations. Additionally, we demonstrate that pathogenic *Vibrio* abundance on macroalgae, oysters, and sediments is each related to distinct environmental conditions.

## INTRODUCTION

Pathogenic *Vibrio* species pose a growing human public health threat in coastal regions worldwide, where warming temperatures have increased infection incidence and expanded their geographic range (1–3). In recent decades, warming waters have led to an increase in *Vibrio* infection risk and a geographic range expansion of pathogenic *Vibrio* presence, with infections now occurring in temperate regions historically considered too cold to support them (4–8).

Much of the foundational research on *Vibrio* ecology has focused on these bacteria as free-living bacterioplankton in the water column or through shellfish (commonly oysters) bioaccumulation of water column *Vibrio* spp. (9, 10). Abundance of *Vibrio* spp. is strongly related to water column conditions like salinity and temperature (10, 11). However, research also indicates that *Vibrio* spp. thrive not only in open water as bacterioplankton but also in association with particles, marine aggregates, organisms, and abiotic substrates such as sediments (12, 13).

Specifically*, Vibrio* spp. associate with planktonic organisms (e.g., phytoplankton or zooplankton), which provide nutrient-rich, stable microhabitats that promote growth and survival in the water column (11, 14–16). Nutrient dynamics often mediate these interactions, with organic matter and nutrient concentrations fueling microalgae growth and, in turn, increasing *Vibrio* abundance (14, 17). *Vibrio* actively colonizes the “phycosphere,” microscale pockets of enriched organic compounds released by phytoplankton and zooplankton, using these hotspots to survive, compete, and often proliferate in the water column (15, 18, 19). *Vibrio* may even show selective attachment based on the composition of the organic matter exudate or through chemically mediated quorum sensing pathways (14). For example, Diner et al. (14) found that pathogenic *Vibrio* preferentially associated with diatoms and copepods that produced chitin. Copepods, in particular, represent a well-documented reservoir and vehicle of transmission for *Vibrio*, enabling dispersal across distances and the ability to persist through less favorable environmental conditions (15, 20). In addition to diverse planktonic hosts, *Vibrio* often attach to marine snow, detritus, fecal pellets, and other particulate organic matter within the water column, providing similar nutrient-rich habitat pockets (19–24). These particle- and planktonic organism-associated lifestyles greatly enhance the survival, persistence, and dispersal of *Vibrio* in coastal systems.

As highly opportunistic bacteria, *Vibrio* spp. can also colonize a wide variety of substrates beyond those in the water column. They have been found in relatively high concentrations on shrimp and crab carapaces (~3.7 to 5.6 log10 CFU per carapace [25]), on commercially important finfish (2.7 to 3.3 log MPN g^−1^ [most probable number method] in both farmed fish like red drum and European sea bass and wild caught fish like parrotfish and grouper [26]), and even on the surface of a range of plastic debris (27). In fact, a recent study found that pathogenic *Vibrio* spp. concentration was three “log-times” higher in the sediments compared to the water column in the Baltic Sea (28).

While microalgae and planktonic hosts are well-known reservoirs for *Vibrio*, the role of macroalgae (i.e., seaweeds) as substrates for pathogenic *Vibrio* is less understood and represents an emerging area of *Vibrio* ecology (29–32). Macroalgae can have a complex surface structure, are rich in organic matter and exudates, and may provide ideal conditions for microbial colonization and persistence (33, 34). Previous work has shown that macroalgae can host diverse microbial communities, including pathogenic *Vibrio* species (29, 32, 35–37), and may even facilitate their growth by supplying accessible nutrients. Therefore, macroalgae may act not only as a passive substrate but also as an active microbial hotspot that buffers *Vibrio* spp. populations from changing water column conditions.

These biotic and abiotic habitats may serve as reservoirs or even proliferation zones for pathogenic *Vibrio* spp. Importantly, the predictive factors traditionally used to explain pathogenic *Vibrio* spp. abundance in the water column may not be as useful for predicting abundance in these other substrates. Predictive models of *Vibrio* spp. risk often rely on temperature and salinity to predict *Vibrio* under the assumption of a bacterioplankton lifestyle (2, 38–40). Yet these same factors are not always good predictors of substrate-associated pathogenic *Vibrio*, where host biology strongly influences abundances (11, 15, 41). Instead, a range of other parameters including organic matter content (42, 43), substrate type, particulate nutrient availability (3, 17), and biofilm formation have all been shown to enhance environmental persistence and stress tolerance of *Vibrio* spp. (24, 41).

This distinction between water column bacterioplankton drivers of abundance highlights the importance of incorporating substrate-specific environmental parameters into *Vibrio* spp. abundance and risk models to improve our understanding of both *Vibrio* spp. ecology and public health implications in a changing environment.

As water temperatures warm, pathogenic *Vibrio* spp. abundance and thus infection risk will continue to rise. Despite this, little is known about how environmental drivers shape *Vibrio* spp. abundance and pathogenicity across different substrate types or how substrate-specific habitat characteristics and importance of environmental factors can influence *Vibrio* spp. ecology (14, 42). Given this, it is both an interesting ecological question and a public health necessity to understand what substrates act as reservoirs for pathogenic *Vibrio* spp. and what environmental conditions favor their growth on these different substrates.

This study investigates the abundance and screens for the presence of pathogenicity-associated genes, which serve as indicators of potentially pathogenic strains of *V. vulnificus* and *V. parahaemolyticus*, in a temperate estuary, across three distinct substrate types: macroalgae, sediment, and oysters. We focus on these two pathogenic *Vibrio* because of their occurrence in coastal waters and their public health impact. *V. vulnificus* causes severe wound infections and septicemia, with a case fatality ranging from 20% to 50%, while *V. parahaemolyticus* is the leading cause of seafood-borne gastroenteritis in the United States (38, 44, 45). Both species of *Vibrio* constitute serious human health risks worldwide, with global models estimating non-cholera *Vibrio* infections at around 0.5 million people per year (1). Specific global estimates for each species remain more difficult to pinpoint due to the differences in country surveillance programs and diagnostic procedures (1, 38, 44, 46–51).

Here, we first compare *V. vulnificus* and *V. parahaemolyticus* abundance and the presence of virulence genes across substrates and then identify the key environmental variables that are associated with *V. vulnificus* and *V. parahaemolyticus* abundance on each substrate. By integrating ecological, microbiological, and statistical modeling approaches, this work addresses a key gap in understanding the complex habitat dynamics and environmental influences of *V. vulnificus* and *V. parahaemolyticus* in coastal systems under change.

## RESULTS

### Sampling overview

**Fig 1:**
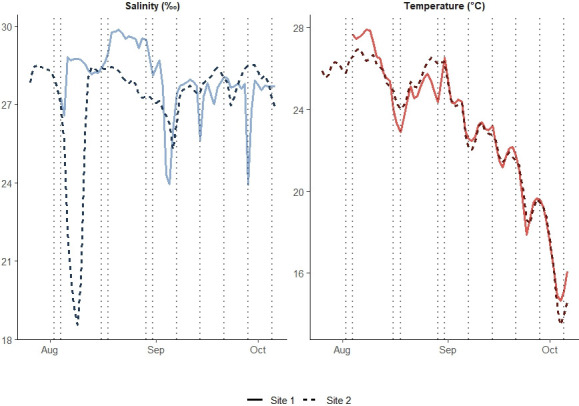
Continuous salinity and temperature data were collected at 1 m depth using Star-ODDI (Garðabær, Iceland) loggers deployed at each site for part of the sampling period (August–October). Colored lines indicate daily measured values for salinity and temperature at each site (Site 1 shown in solid lines and Site 2 in dashed lines). Dotted vertical lines show the specific dates where sites were sampled.

We quantified *V. vulnificus* and *V. parahaemolyticus* abundance on three different substrates at two oyster (*Crassostrea virginica*) farms in the coastal waters of Rhode Island, USA (see Site descriptions in the supplemental material). We collected samples of macroalgae, oysters, and sediment in triplicate bi-monthly from June to October 2022. Additionally, at each sample collection, we also measured common surface water quality parameters (Table 1). Mid-way through the study period, we were able to deploy sondes at each site that logged salinity and temperature (Fig. 1). Average mean temperature and salinity were similar during sample collection (Table 1), though the sonde data highlight differences in salinity patterns between the two sites (Fig. 1). Dissolved inorganic nitrogen (DIN) and phosphorus concentrations as well as total suspended solids (TSS) were higher at site 1 (Table 1).

**TABLE 1:** Site characteristics during this study (bi-monthly at each site from June to October 2022)^*a*^

Site name	Site 1	Site 2
Temperature (°C)	21.8 (16.7–26.7)	20.9 (14.9–23.6)
Salinity (‰)	30.9 (27–33.0)	30.6 (26.0–32.5)
Dissolved oxygen (mg L^−1^)	6.4 (4.4–7.9)	6.5 (5.0–7.8)
Ammonium (NH_4_^+^) (µmol L^−1^)	2.2 (0.9–5.8)	2.0 (1.4–3.4)
NOx (nitrite [NO_2_^−^] + nitrate [NO_3_^−^]) (µmol L^−1^)	1.7 (0.02–4.4)	0.4 (0.13–0.88)
Dissolved inorganic nitrogen (µmol L^−1^)	3.9 (1.0–7.7)	2.4 (1.7–3.9)
Dissolved inorganic phosphorus (µmol L^−1^)	2.1 (0.8–2.9)	0.7 (0.42–1.1)
Chlorophyll-a (µg L^−1^)	2.3 (0.5–3.4)	2.7 (0.86–10.6)
Phaeophytin (µg L^−1^)	3.3 (0.62–7.8)	2.2 (1.2–4.2)
TSS (mg L^−1^)	1.3 (0.3–3.9)	0.9 (0.3–1.7)

^*a*^ Mean (min-max) is reported for each parameter as measured on the day of pathogenic *Vibrio *spp. sample collection.

### Vibrio spp. quantification

**Fig 2:**
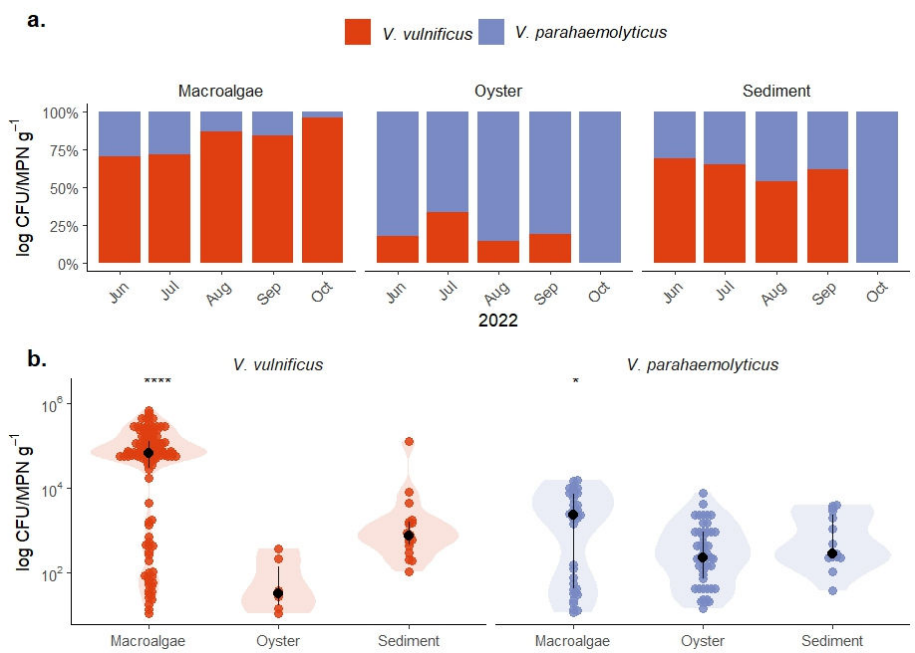
(**a**) Stacked bar plot representing the proportional abundance of *V. parahaemolyticus* (purple) and *V. vulnificus* (orange) across three substrate types (macroalgae, oysters, and sediment) averaged for every month of the study (June–October 2022). Each bar shows the relative proportion of each species to the combined total. (**b**) Log-transformed bacterial abundance counts of *V. vulnificus* (orange) and *V. parahaemolyticus* (purple) across three substrate types (macroalgae, oysters, and sediment) across the study period. Macroalgae exhibited higher abundances of both *V. vulnificus* (****P < 0.00001) and *V. parahaemolyticus* (*P < 0.05) compared to both oysters and sediment. *Vibrio* abundance was enumerated using the MPN method in oysters and CFU counts in sediment and macroalgae. MPN g⁻¹ and CFU g⁻¹ are treated as equivalent units for microbial quantification, with MPN having a lower limit of detection, while CFU counts provide finer scale information. See Tables S1 and S2 for detailed sample counts.

Over the study period, we quantified *V. vulnificus* and *V. parahaemolyticus* abundance on six different genera of macroalgae (*Codium, Enteromorpha, Fucus, Gracilaria, Polysiphonia, Saccharina, and Ulva,* Table S1). The study period median macroalgae abundance for *V. vulnificus* was 5.03 × 10^4^ CFU g⁻¹ (interquartile range [IQR] of 2.81 × 10^4^), and for *V. parahaemolyticus*, it was 1 × 10^1^ CFU g⁻¹ (IQR of 2.51 × 10^1^) (Fig. 2a). We observed the maximum *V. vulnificus* abundance on *Fucus* spp. (6.60 × 10^5^ CFU g⁻¹) and the maximum *V. parahaemolyticus* on *Ulva* spp. (1.56 × 10^4^ CFU g⁻¹). *V. vulnificus* abundance varied significantly across macroalgae genera, with the differences driven by *Fucus,* which had higher abundances compared to *Codium* (*P* = 0.0026), *Enteromorpha* (*P* = 0.0018), *Gracilaria* (*P* = 0.0062), and *Ulva* (*P* = 0.0267). In contrast, *V. parahaemolyticus* abundance did not differ significantly across macroalgae genera.

Oysters had maximum abundances of 3.60 × 10^2^ MPN g⁻¹ for *V. vulnificus* and 7.48 × 10^3^ MPNg⁻¹ for *V. parahaemolyticus*. Oyster *V. vulnificus* had a median of 1.12 × 10^1^ MPN g⁻¹ and an IQR of 2.66 × 10^1^. *V. parahaemolyticus* had a median of 2.10 × 10^2^ MPN g⁻¹ with an IQR of 3.59 × 10^1^. MPN, or most probable number, is a statistics-based measurement of bacterial abundance, while CFUs are calculated via direct plate counting. These measurements are considered equivalent to one another and can be directly compared (52).

Sediment had a maximum CFU g⁻¹ count of 1.24 × 10^5^ for *V. vulnificus* and 3.89 × 10^3^ for *V. parahaemolyticus*. Sediment *V. vulnificus* had a median of 1 × 10^1^ and an IQR of 2.21 × 10^2^. *V. parahaemolyticus* had a median of 1 × 10^1^ with an IQR of 7.29 × 10^1^.

We found no significant differences in *V. vulnificus* or *V. parahaemolyticus* abundance between the two different sites for any sample type (Kruskal-Wallis, *P* > 0.05). Because no significant differences were detected between the two sites, data were combined for subsequent analyses examining how *V. vulnificus* and *V. parahaemolyticus* abundance varied between substrates. We found that *V. vulnificus* (*P* < 0.00001) and *V. parahaemolyticus* (*P* < 0.05) abundance was significantly higher on macroalgae compared to oysters and sediments (Fig. 2a). Sediment tended to have higher *V. vulnificus* and *V. parahaemolyticus* abundance compared to oysters, though not significantly so.

Macroalgae had significantly higher amounts of *V. vulnificus* (*P* < 0.00001) and *V. parahaemolyticus* (*P* < 0.05) compared to oysters and sediment (Fig. 2b). *V. vulnificus* was detected at much lower levels in oysters compared to sediment and macroalgae. Oysters had the largest nondetection rate at 50%, with sediment and macroalgae having nondetection rates of 47% and 20%, respectively.

Neither *V. vulnificus* nor *V. parahaemolyticus* abundance varied significantly with time for each substrate across the study period (*P* > 0.05). However, the relative proportion of *V. vulnificus* compared to *V. parahaemolyticus* varied greatly (Fig. 2b). Notably, across all months, macroalgae continually hosted a higher proportion of *V. vulnificus*, with percentages reaching upward of 75% of the total culturable *Vibrio* population. Conversely, oysters were dominated by *V. parahaemolyticus* abundance throughout the study period, with percentages often close to 80%. Sediment showed a general trend of higher percentages of *V. vulnificus*, except for the final month of the study, where only *V. parahaemolyticus* was detected.

To assess whether *Vibrio* abundances on substrates were related to one another, we compared each species across sample types. That is, we wanted to test if, for example, pathogenic *Vibrio* abundance on macroalgae correlates to higher or lower abundance in oysters or the sediment. No significant correlations were found between substrate types for either *Vibrio* species, indicating that in this study, the presence on one substrate did not directly indicate the presence on another. However, a significant positive correlation was observed between *V. parahaemolyticus* and *V. vulnificus* within macroalgae samples (*P* = 0.0027, Fig. S2), suggesting potential co-occurrence within that substrate.

### Pathogenic gene presence across substrates

The presence of the *toxR* gene (marker for confirming *V. vulnificus*) varied among the substrate types. The detection rate of *tlh*, the gene marker for confirming *V. parahaemolyticus*, was similar across substrates, with macroalgae being the highest at 60%, sediment at 53%, and oysters at 50%. In each sample type, a proportion of all samples taken was considered non-detect, with no growth in culture methods and therefore no subsequent qPCR.

**Fig 3:**
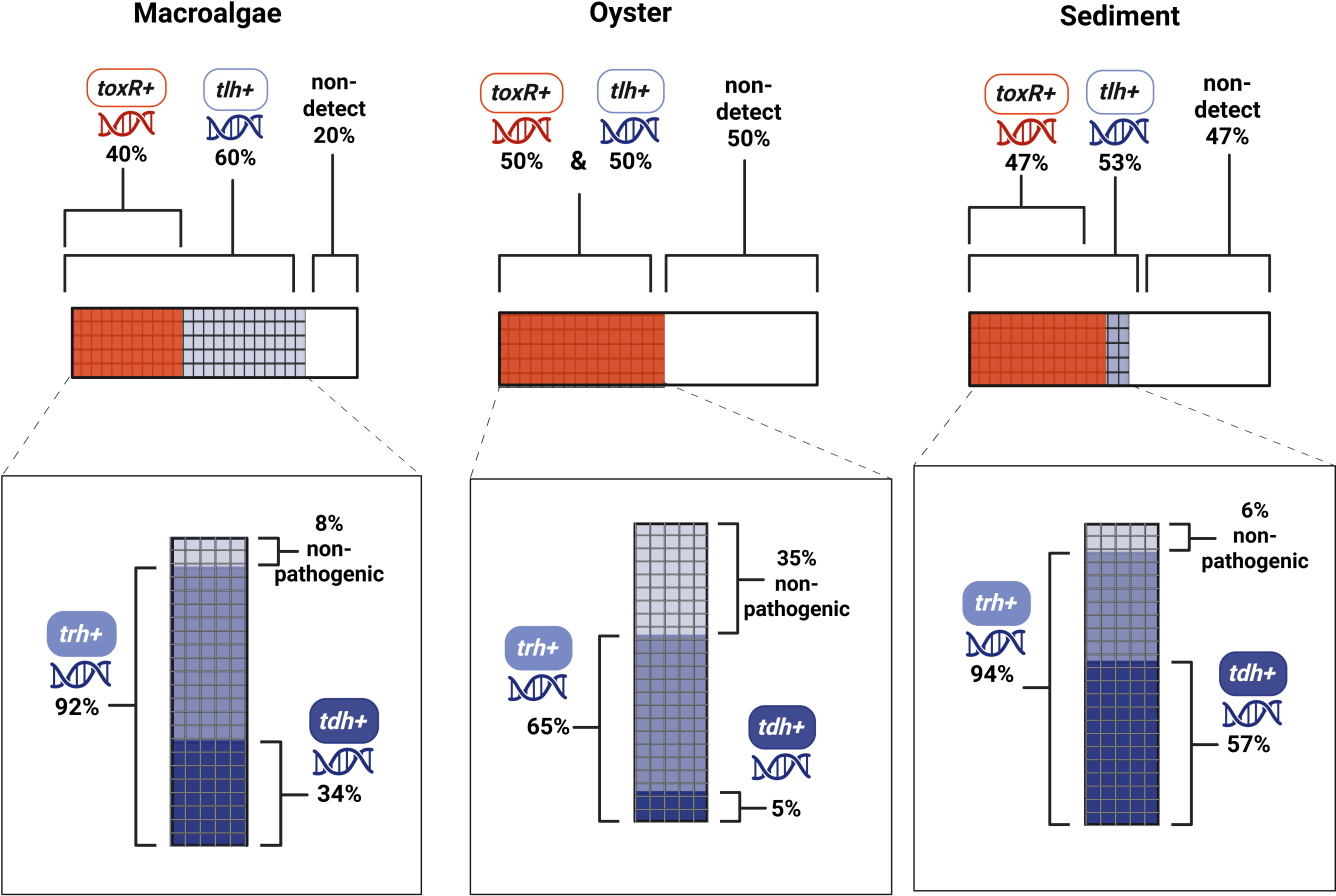
*Vibrio* gene detection via qPCR across macroalgae, oyster, and sediment samples. The top panels show the proportion of samples from each substrate classified as either non-detect (white; no colony growth on CHROMagar or detection via the MPN method and therefore not tested via qPCR); *tlh*^+^ (purple checkerboard), indicating the presence of *V. parahaemolyticus*; or *toxR*^+^ (orange), indicating the presence of *V. vulnificus* (see Table S3 for further details). In all cases, *toxR*^+^ samples were also *tlh*^+^. The bottom panels display the *tlh*^+^ samples, which underwent further testing for pathogenicity-associated genes *trh* and *tdh*. Notably, *tdh* was always detected alongside *trh*, suggesting the presence of a potentially highly virulent *V. parahaemolyticus* strain. The proportions of tlh^+^ samples classified as pathogenic (harboring *trh* and/or *tdh*) versus nonpathogenic (no detection of *trh* and/or *tdh*) are indicated for each substrate.

In this study, all samples that were positive for *toxR* were also positive for *tlh* (Fig. 3; Table S3). This is consistent with previous findings for macroalgae measured at three different, non-oyster farm sites in coastal Rhode Island (32). It aligns with the general idea that the two *Vibrio* species studied here often overlap in environmental niches (9). Among the samples positive for *tlh* (*V. parahaemolyticus*), the proportion that had one or both pathogenic gene markers varied by substrate type. In macroalgae, 92% of *tlh*+ samples are considered pathogenic due to the detection of at least one of the pathogenicity genes (*tdh*/*trh*). Among these confirmed pathogenic *V. parahaemolyticus*, 34% had both pathogenicity genes present, indicating the potential presence of a highly virulent strain of *V. parahaemolyticus* (53, 54). In sediment samples, 94% of *V. parahaemolyticu*s were considered pathogenic, with 57% of those having both pathogenic gene markers. Oyster samples showed a lower percentage of pathogenic detections, with 65% that were considered pathogenic and only 5% that contained both pathogenic genes.

### *Vibrio* abundance and environmental conditions

*V. vulnificus* on macroalgae was most influenced by chlorophyll-a, dissolved inorganic phosphate (DIP), phaeophytin, salinity, temperature, and TSS (Table 2). This model was weak, only explaining 12% of the variance. Macroalgae *V. parahaemolyticus* abundance was best explained by the combination of chlorophyll-a, DIP, salinity, temperature, and TSS, with 47% of the variance explained in this model (Akaike’s information criterion [AIC] = 822).

**TABLE 2:** Summary of generalized additive models (GAMs) predicting *V. parahaemolyticus* and *V. vulnificus* log-abundances across three sample types (macroalgae, sediment, and oyster)^*a*^

Sample type ~ species	Model parameters	AIC	Variance explained
Macroalgae ~ VP	vp_count ~ s(chla) + s(dip) + s(sal) + s(temp) + s(tss) + 1	822	47%
Macroalgae ~ VV	vv_count ~ s(chla) + s(dip) + s(phaeo) + s(sal) + s(tss) + 1	3,219	12%
Sediment ~ VP	vp_count ~ s(chla) + s(din) + s(phaeo) + s(sal) + s(temp) + s(tss) + 1	319	51%
Sediment ~ VV	vv_count ~ s(chla) + s(din) + s(dip) + s(phaeo) + s(sal) + s(temp) + 1	305	87%
Oyster ~ VP	vp_log_count ~ s(chla) + s(din) + s(dip) + s(phaeo) + s(temp) + s(tss) + 1	118	27%
Oyster ~ VV	vv_log_count ~ s(temp) + 1	63	35%

^*a*^ Models include environmental predictors (e.g., chlorophyll-a, nutrients, temperature, salinity, and TSS), AIC values, and percent variance explained.

The abundance of *V. vulnificus* in oysters was best explained by a model that contained only one parameter: temperature. The single-variable model accounted for 35% of the variance seen in *V. vulnificus* abundance in oysters (AIC = 63). Oyster *V. parahaemolyticus* abundance was best explained by chlorophyll-a, DIN, DIP, phaeophytin, temperature, and TSS, with 27% of the variance explained (AIC = 118).

In sediment, *V. vulnificus* showed the strongest model fit, with chlorophyll-a, DIN, DIP, phaeophytin, salinity, and temperature explaining 87% of the variance in abundance within sediments (AIC = 305). *V. parahaemolyticus* was influenced by chlorophyll-a, DIN, phaeophytin, salinity, temperature, and TSS. This model explained 51% of the variance (AIC = 319).

## DISCUSSION

This study provides new insights into the ecology of *V. parahaemolyticus* and *V. vulnificus* by examining their abundances across three substrates, macroalgae, sediment, and oysters, sampled at two oyster farms in a temperate estuary. We show that macroalgae are a host of pathogenic *Vibrio*, consistent with our previous work at different sites in the same estuary (32), and more importantly, we highlight that the abundance of pathogenic *Vibrio* on different substrates is related to different environmental conditions.

### *Vibrio* spp. abundance across substrates

Macroalgae hosted significantly higher abundances (log₁₀ CFU g⁻¹ wet weight) of both *V. parahaemolyticus* and *V. vulnificus* relative to oysters (log₁₀ CFU g⁻¹ tissue) and sediments (log₁₀ CFU g⁻¹ dry weight) throughout the sampling period. These findings support the growing body of literature demonstrating that macroalgae can provide a nutrient-rich and protective microhabitat helpful to *Vibrio* spp. persistence and growth (29, 32, 35–37, 41). Other studies have also documented pathogenic *Vibrio* colonization of macroalgae tissue, including *Gracilaria* spp. along the U.S. southeastern coast and multiple macroalgae species in the Kii channel of Japan, highlighting the potential widespread—yet often overlooked—importance of this substrate (29, 36, 37).

In contrast, oysters had the lowest observed abundance of *V. vulnificus*, with larger proportions of *V. parahaemolyticus* overall. This pattern aligns with the established knowledge of oyster filter-feeding behavior, which accumulates planktonic bacteria such as *V. parahaemolyticus*, especially during warmer months (55, 56). The *Vibrio* abundances in oysters found in this study are also consistent with previous findings from Narragansett Bay (57, 58). Additionally, the generalist metabolic nature of *V. parahaemolyticus* and its affinity for free-living or microparticle association likely contribute to the greater prevalence in oyster tissues relative to *V. vulnificus* in this study (44, 59). Even so, oyster-associated *Vibrio* can vary across systems, and oyster shell-associated communities may differ from those within the oyster tissue (60).

Comparatively, sediment supported high abundances of *V. vulnificus*, often comprising more than 50% of the culturable *Vibrio*. Similar trends have been observed along the U.S. Gulf Coast, where *V. vulnificus* persisted in sediment during winter months when concentrations in oysters and water were undetectable (13, 61). Sediment may provide organic matter, reduced competition, and buffered environmental conditions, representing an ecologically favorable and stable habitat for *Vibrio* (13).

The comparatively higher abundance of *V. vulnificus* in sediment and macroalgae relative to oysters in this study is consistent with its physiological ecology. *V. vulnificus* often exhibits greater environmental sensitivity and a stronger preference for surface attachment and biofilm formation, particularly under suboptimal environmental conditions (24, 62, 63). Biofilms enhance survival by offering protection from predation, UV exposure, and nutrient scarcity (24). When these biofilms form on substrates rich in organic matter, like macroalgal tissue or nutrient-rich sediment, they may offer further metabolic and ecological advantages (41). These survival strategies may help explain why *V. vulnificus* populations associated with macroalgae and sediment were consistently higher than those in oysters in this study, highlighting the need to further investigate substrate-specific niches of a variety of different species of *Vibrio* and their potential habitats.

### Pathogenicity across substrates

Understanding the ecology and public health implications of *Vibrio* spp. requires evaluating the distribution of pathogenic genotypes. In this study, we screened for the *toxR* gene as an indication of *V. vulnificus*. Although *toxR* is widely used for species identification within Vibrionaceae, its presence alone does not fully determine virulence, though its presence is widely accepted as a marker for general pathogenicity (9, 49, 64). For *V. parahaemolyticus*, the presence of the *tdh* (thermostable direct hemolysin) and *trh* (TDH-related hemolysin) genes is considered reliable indicators of virulence, despite the broader complexity of pathogenicity, which includes regulatory pathways such as quorum sensing and type III secretion systems (44, 65, 66). Strains of *V. parahaemolyticus* lacking these genes may still cause harm, including as pathogens of marine organisms such as shrimp (25).

### Environmental controls across substrates

To better understand the ecology of *V. vulnificus* and *V. parahaemolyticus*, in this coastal system, we modeled species-specific responses to environmental conditions for each substrate type. Temperature and salinity are often related to *Vibrio* dynamics, especially for free-living *Vibrio* in the water column (40, 42, 67). In this study, temperature alone explained nearly 35% of the variation in *V. vulnificus* abundance in oysters. We hypothesize that this is likely due to the direct accumulation of waterborne bacteria through filter-feeding (56).

In contrast, macroalgae and sediment-associated *Vibrio* populations were influenced by a broader suite of environmental variables, including nutrient (e.g., DIP and DIN) concentrations and indicators of organic matter (e.g., phaeophytin and TSS). These findings align with the concept that substrate-specific microhabitats, shaped by biogeochemical conditions, may affect *Vibrio* growth independently of classic water column drivers (68–70). Thus, effective monitoring of *Vibrio*-related human health risk should account for drivers of substrate-associated populations, not just planktonic ones.

To improve predictions of when, where, and why pathogenic *Vibrio* bacteria proliferate, it is essential to investigate not only the planktonic populations and water column conditions but also other substrate-associated reservoirs and the conditions that affect them. Incorporating diverse habitats and environmental drivers into surveillance and forecasting strategies will be growing in importance as the climate continues to change.

## MATERIALS AND METHODS

### Sample collection

We collected samples twice monthly from June 2022 to October 2022. This temporal scale was designed to capture both the growing season of macroalgae as well as the warmest yearly temperatures in RI, which should facilitate *Vibrio* spp. growth (71, 72). Our 5-month sampling period captures the primary *Vibrio* growing season, but longer-term monitoring would be required to evaluate interannual environmental controls. For each sampling event, we collected the three dominant genera of macroalgae directly off aquaculture infrastructure at each site, along with triplicate sets of 10 oysters and triplicate surface sediment (top 1 cm) at each oyster farm. Upon collection, macroalgae, sediment, and oysters were immediately put on ice and processed the same day (~3 h of collection). The aquaculture farms were located in shallow near-shore sites in Narragansett Bay (see Site descriptions in the supplemental material).

### Macroalgae and sediment laboratory processing

We weighed out macroalgae samples to a representative 10-g sample, combined with 100 mL phosphate-buffered saline (PBS), and shaken for 5 min to disrupt the biofilm *Vibrio* forms on the macroalgae. All macroalgae samples were plated on thiosulfate-citrate-bile salts-sucrose (TCBS) medium for total *Vibrio* counts and also on CHROMagar *Vibrio* medium (CHROMagar) to determine counts for *V. parahaemolyticus* and *V. vulnificus*. Sediment was collected from the top 1 cm of the benthos using a sterile modified 60-mL syringe as a corer with a 25 mm diameter. Samples were kept on ice and processed within 3 h of collection (73). Sediment samples were combined with equal parts of PBS and weight (e.g., 15 g sediment and 15 mL PBS (29). The slurry was serially diluted using PBS and spread across both agar types. To control for the differences in the initial water content of the sediment, 2 mL of each sediment slurry was filtered onto pre-weighed filters, dried in a 60°C oven for a minimum of 48 h, and reweighed. The dry weight mass was used for later calculations of *Vibrio* concentrations per gram of dry weight. This same technique was used to account for the variation in macroalgae water content. The initial 10 g used in sample analysis was dried in pre-weighed tins and reweighed to determine the dry weight. All macroalgae and sediment samples plated on CHROMagar and TCBS plates were incubated in a 37°C incubator according to the manufacturer’s instructions. The CFUs were then enumerated on each plate to determine the presumptive *Vibrio* concentrations. Isolated colonies (as many were present) were removed from the plates with sterile loops and then extracted using the Qiagen power-water DNA extraction kit for later qPCR. CFUs were standardized to sample dry mass and expressed as CFU g⁻¹. For macroalgae, values were calculated as CFU per gram dry weight. Sediment abundances were expressed as CFU per gram dry weight following oven-drying at 60°C to constant mass. When applicable, MPN values were expressed as MPN g⁻¹. All abundance values were log₁₀-transformed prior to statistical analysis (74).

### Oyster laboratory processing

We collected triplicate sets of oysters (*n* = 10 individuals) at each sampling event to be analyzed. Oysters were grown on the aquaculture farms using rack and bag set-up (site 1) or floating bags (site 2) (75, 76). Upon arrival at the lab, we carefully cleaned oyster shell exteriors to remove biofouling. We then sterility shucked the entire tissue content (meat and hemolymph) and placed it in a blender for homogenization, aligning with the FDA guidelines for MPN enumeration methods (77). This homogenate was serially diluted in phosphate-buffered saline, and then 1 mL of each dilution was added to alkaline peptone water media for incubation at 35°C and 150 rpm for 20 h. The following day, we determined bacterial growth in the tubes by confirming cloudiness of the media (meaning bacteria growth was present). The number of positive tubes at each dilution was compared to the FDA chart to determine the estimated abundance of *Vibrio* spp. in the sample (77). From each positive tube, 1 mL of culture water was sampled and extracted using the Qiagen power-water DNA extraction kit for later qPCR. Only samples over 10 copies are deemed positive according to FDA standards.

### Molecular typing

We first confirmed *Vibrio* species type using qPCR assay protocols created by Scro et al. (58). We confirmed the presence of *V. parahaemolyticus* by using primers for *tlh* that is found in *V. parahaemolyticus* (58, 78, 79) and known qPCR conditions (58, 80). *V. vulnificus* was confirmed by the *toxR* gene, as well as known qPCR conditions (58, 64). After initial identification, we performed another multiplex qPCR assay on any sample positive for *V. parahaemolyticus* to determine the quantity of pathogenic genes. Genes that encode for thermostable direct hemolysin (*tdh*) and thermostable direct hemolysin-related hemolysin (*trh*) are reliable indicators of the virulence of *V. parahaemolyticus* (78, 81). All *V. vulnificus* is considered pathogenic, so no further genetic identification was needed.

### Environmental laboratory processing

We used standard colorimetric techniques and a high-resolution-digital colorimetry on a Seal Auto Analyzer 3 with segmented flow injection to analyze water samples for dissolved inorganic nitrogen and phosphorus (82–84). The detection limits for these analyses were NH_4_^+^ 0.080 µM, NO_x_ 0.013 µM, NO_3_^−^ 0.013 µM, NO_2_^−^ 0.006 µM, and PO_4_^3−^ 0.010 µM. We analyzed filters for chlorophyll-a and pheophytin using standard acetone extraction and a Turner Designs TD-700 fluorometer (Sigma-Aldrich, St. Louis, MO, USA) calibrated with pure chlorophyll-a (82, 83, 85, 86).

Along with the macroalgae samples, we collected water samples for concentrations of dissolved inorganic nitrogen and phosphorus, chlorophyll-a and phaeophytin, and total suspended solids. In the field, we filtered duplicate water samples using a 60 mL acid-washed polypropylene syringe with glass fiber filters (Whatman GF/F, 0.70 micron pore size) into 30 mL acid-washed and deionized water leached polyethylene bottles. The filter samples were stored on ice in a dark cooler until return to the lab and then were stored in a freezer (−20°C) until analysis for DIN (NH_4_^+^, NO_2_^−^, and NO_3_^−^), and DIP (85). Chlorophyll-a and phaeophytin measurements were also collected in duplicate at each site using deionized water-cleaned 60-mL polypropylene syringes. Filters were stored in the dark, on ice, until return to the lab, and then at −80 °C until analysis. During each macroalgae collection, we also measured water column temperature, salinity, and dissolved oxygen using a HACH sensor (LDO101).

### Statistical analyses

We used a random forest model to identify the most important environmental variables associated with *Vibrio* spp. abundance among sample types. We used 500 trees with default hyperparameters, with variable importance being assessed based on the increase in mean squared error (%IncMSE), which measures how much the accuracy of the model decreases when a variable is randomly changed, with higher values indicating that removing the predictor variable significantly reduces the model performance, meaning that it is an influential predictor (Fig. S1) (87, 88). These metrics are widely used in ecological and microbial studies to determine key environmental drivers without linear relationships (89, 90). Separate models were run for each combination of *Vibrio* species and substrate type and included all relevant environmental variables that did not covary, determined by Spearman correlation analysis (Fig. S2). The results of the random forest models were used to guide the variable selection and creation of subsequent GAMs. To fully explore the nonlinear relationships between *Vibrio* abundance and environmental conditions while associated with different substrates, we created GAMs using the *mgcv* package in R (91). Before selecting the error distribution for the GAMs, we first evaluated the distribution and properties of the count data for each *Vibrio* species. We compared the mean and variance of the counts and conducted overdispersion tests on Poisson models. In all cases (each individual GAM data subset), the variance substantially exceeded the mean, indicating a large overdispersion of the data. We compared Poisson and negative binomial fits using AIC and found that the negative binomial models consistently gave a better model output and fit to the data. This approach follows the recommended procedure for identifying the proper distribution for ecological data (see Table S4 for further information) (92–94). The count data of each *Vibrio* species on substrate type were modeled using individual GAMs adjusted with a negative binomial family dispersion to account for the overdispersion in the data, which is common among bacterial count data (94). The initial GAMs included all the environmental variables that were included in the first random forest models. To assess model performance and find the best GAM, we used *MuMIn:dredge (*95) to perform automated permutation and analysis of the models based on AIC (96). The lowest AIC value model was selected for interpretation. Diagnostic checks were run using the *gratia* and *mgcv* packages for the negative binomial GAMs, which included residual analysis, concurvity diagnostics, and overdispersion and zero inflation checks (97, 98). Overall, random forest models were first used as a nonparametric way to assess variable importance compared across substrate and *Vibrio* species. The next step of creating GAMs provided us with more interpretable and statistically robust measures of environmental effects on *Vibrio* abundance.

## Data Availability

Data are available at https://doi.org/10.6084/m9.figshare.30904115.

## SUPPLEMENTAL MATERIAL

Supplemental material aem.02580-25-s0001.docx Site descriptions, Tables S1 to S4, and Fig. S1 and S2.

ASM does not own the copyrights to Supplemental Material that may be linked to, or accessed through, an article. The authors have granted ASM a non-exclusive, world-wide license to publish the Supplemental Material files. Please contact the corresponding author directly for reuse.
