# Supplementary material for: Examining pathogenic Vibrio abundance and environmental controls across macroalgae, sediment, and oysters
Source: Appl Environ Microbiol. 2026 Apr 17;92(5):e02580-25. doi: 10.1128/aem.02580-25 (PMC13188903; doi:10.1128/aem.02580-25)
Supplement: Supplemental material — Site descriptions, Tables S1 to S4, and Fig. S1 and S2. [file aem.02580-25-s0001.docx]

Supplementary Materials for: **Examining Pathogenic *Vibrio* Abundance and Environmental Controls Across Macroalgae, Sediment, and Oysters**

AUTHORS: Alexandra Geisser^a^#, Abigail K. Scro^b^, Roxanna Smolowitz^b^, Robinson W. Fulweiler^a,c^

^a^Dept. of Biology, Boston University, Boston, MA, USA

^b^Aquatic Diagnostic Laboratory, Center for Economic and Environmental Development, Roger Williams University, Bristol, RI, USA

^c^Dept. of Earth & Environment, Boston University, Boston, MA, USA

Running Head: *Examining Pathogenic Vibrio Across Substrates*

#Address correspondence to Alexandra Geisser, ageisser@bu.edu

| **Location** | **Description** |
| --- | --- |
| Page 2 | Site descriptions |
| Page 3 | Supplementary Table 1 |
| Page 3 | Supplementary Table 2 |
| Page 4 | Supplementary Table 3 |
| Page 5 | Supplementary Table 4 |
| Page 6 | Supplementary Figure 1 |
| Page 7 | Supplementary Figure 2 |

**Site Descriptions:**

Site 1 is located within a shallow, coastal lagoon in the southern shore of Rhode Island (RI, USA). This lagoon is tidally influenced, with a tidal range of 0.4 m and residence time of ~ 2 days (Rozier, 2015; Russell, 2024). The area is surrounded by a watershed of mixed land-use including undisturbed areas, high and medium impact residential areas, and pasture land (Rozier, 2015; Russell, 2024). Water quality has been a long-term concern in this lagoon because of excess nutrients causing eutrophication and because of bacterial contamination (Fofonoff & Lee, 1992). During this study water temperature ranged between 16.7 and 26.7 °C with salinity reaching a maximum of 33. Dissolved inorganic nitrogen (DIN) concentrations ranged between 1.0 and 7.7 mmol L^-1^ and ammonium was typically the primary component of DIN (Table 1). Chlorophyll-a and total suspended solid (TSS) concentrations were also variable over the study period, with a range of 0.5 to 3.4 ug L^-1^ to 0.3 to 3.9 mg L^-1^, respectively.

Site 2 is a semi-protected, shallow cove in Narragansett Bay (RI, USA) proper. Narragansett Bay proper is 234 km^2^ with a tidal range of 1.2 m and a residence time of 26 days (Nixon et al., 2009). The habitats surrounding this cove include sandy and muddy habitats, with a lightly developed coastline. During this study water temperature ranged between 14.9 and 23.6 °C with salinity reaching a maximum of 32.5. Dissolved inorganic nitrogen (DIN) concentrations ranged between 1.7 and 3.9 mmol L^-1^ and ammonium was typically the primary component of DIN (Table 1). Chlorophyll-a and total suspended solid (TSS) concentrations were also variable over the study period, with a range of 0.86 to 10.6 ug L^-1^ to 0.3 to 1.7 mg L^-1^, respectively.

**Supplementary Table 1**. Summary of the number and genera of macroalgae samples collected at each site.

| **Macroalgae Genera** | **Site 1** | **Site 2** |
| --- | --- | --- |
| *Codium spp.* | 30 | 18 |
| *Enteromorpha spp.* | 0 | 12 |
| *Fucus spp.* | 18 | 12 |
| *Gracilaria spp*. | 3 | 9 |
| *Polysiphonia spp*. | 9 | 3 |
| *Saccharina spp.* | 0 | 3 |
| *Ulva spp.* | 18 | 45 |

**Supplementary Table 2**. Summary of the number macroalgae, oyster, and sediment samples collected, separated by site.

|  | **Macroalgae** | **Sediment** | **Oysters** | **Total** |
| --- | --- | --- | --- | --- |
| Site 1 | 78 | 30 | 30 | 138 |
| Site 2 | 102 | 32 | 33 | 167 |

**Supplementary Table 3**. Summary of macroalgae, oyster, and sediment samples collected and screened for *Vibrio* species and associated genetic markers for both sites combined. Values represent the percentage (and number) of samples within each substrate in which each target was detected. Gene detections are not mutually exclusive, that is individual samples may contain multiple *Vibrio* species and/or virulence-associated genes. As a result, percentages within a substrate may exceed 100%. Non-pathogenic *V. parahaemolyticus* are samples lacking detection of tdh and trh.

|  | **Macroalgae**  (total= 180) | **Oyster**  (total= 62) | **Sediment**  (total= 63) |
| --- | --- | --- | --- |
| **Non-detect** | 20%  (n=36) | 50%  (n=31) | 47%  (n=29) |
| *V. vulnificus****- toxR+*** | 40%  (n=72) | 50%  (n=31) | 47%  (n=29) |
| *V. parahaemolyticus****- tlh +*** | 60%  (n=108) | 50%  (n=31) | 53%  (n=34) |
| *V. parahaemolyticus***- non pathogenic** | 8%  (n=9) | 35%  (n=11) | 6%  (n=2) |
| *V. parahaemolyticus* ***tdh +*** | 34%  (n=37) | 5%  (n=1) | 57%  (n=19) |
| *V. parahaemolyticus* ***trh +*** | 92%  (n=99) | 65%  (n=20) | 94%  (n=32) |

| **Supplementary Table 4**. GAM model parameters for the best fit model, with distribution family, AIC, and R^2^ adjusted metrics also reported. In all cases, the variance substantially exceeded the mean, and Poisson models were not as well performing as negative binomial models. | | | | | |
| --- | --- | --- | --- | --- | --- |
| **Sample type ~ species** | **Model parameters** | **AIC** | **Distribution Family** | **R^2^ adj.** | **Variance Explained** |
| Macroalgae ~ VP | vp_count ~ s(chla) + s(dip) + s(sal) + s(temp) + s(tss) + 1 | 822.5 | Negative binomial | 0.134 | 47.4% |
| Macroalgae ~ VV | vv_count ~ s(chla) + s(dip) + s(phaeo) + s(sal) + s(tss) + 1 | 3219.1 | Negative binomial | 0.143 | 11.6% |
| Sediment ~ VP | vp_count ~ s(chla) + s(din) + s(phaeo) + s(sal) + s(temp) + s(tss) +  1 | 319.5 | Negative binomial | 0.247 | 51.1% |
| Sediment ~ VV | vv_count ~ s(chla) + s(din) + s(dip) + s(phaeo) + s(sal) + s(temp) +  1 | 305.2 | Negative binomial | 0.268 | 86.6% |
| Oyster ~ VP | vp_log_count ~ s(chla) + s(din) + s(dip) + s(phaeo) + s(temp) +  s(tss) + 1 | 118.7 | Negative binomial | 0.328 | 27.4% |
| Oyster ~ VV | vv_log_count ~ s(temp) + 1 | 63.2 | Negative binomial | 0.305 | 34.9% |


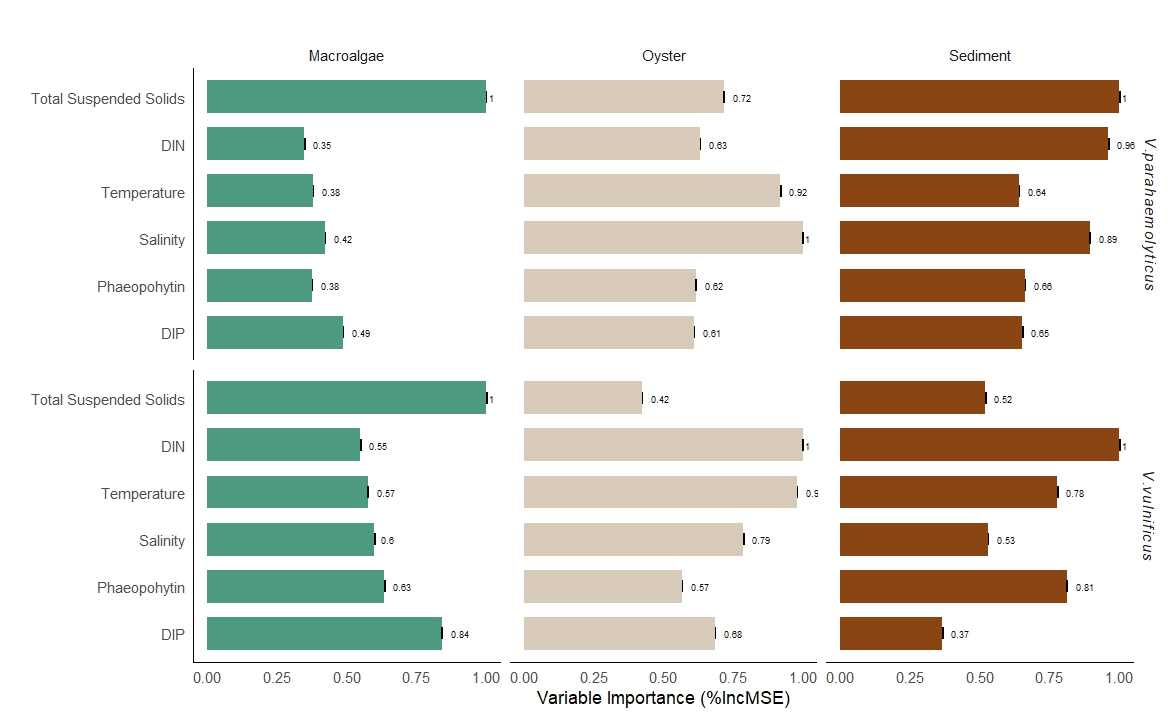


**Supplementary Figure 1.** Random forest models of environmental parameters for each substrate and *Vibrio* species. The graph shows %IncMSE (Increase in Mean Squared Error) as the metric for measuring variable importance, which accounts for the potential nonlinear relationships and is a more robust metric for determining key predictors in a model because it directly measures the model performance. A higher %IncMSE value indicates that the variable is more important for model accuracy.


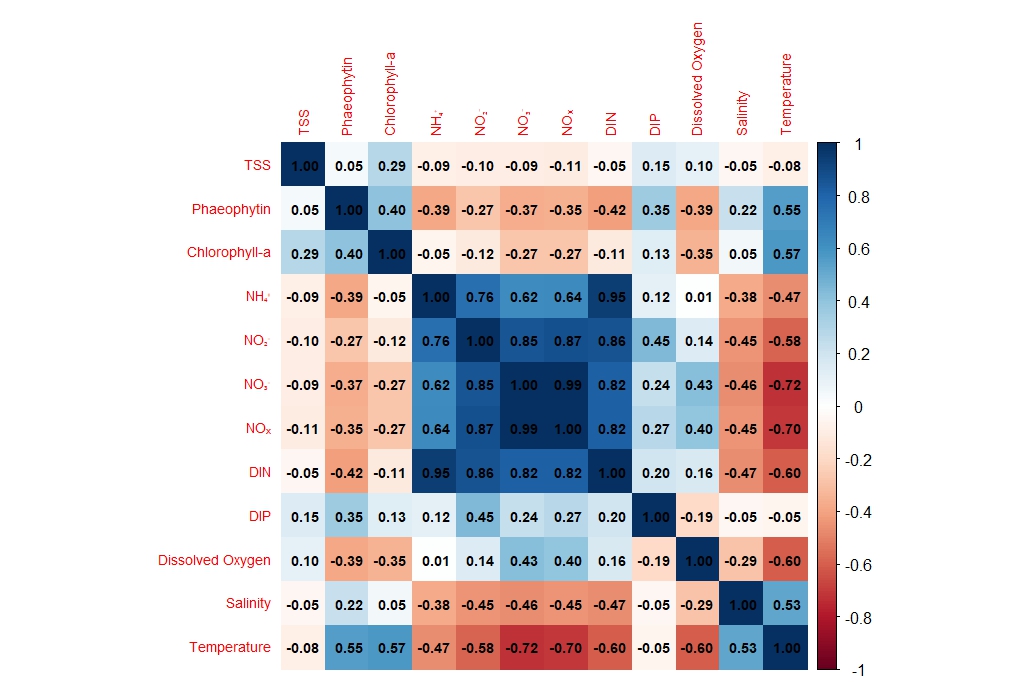


**Supplementary Figure 2**. Spearman correlation analysis of environmental variables to determine covariance. Correlation analysis was used to select the environmental variables that did not co-vary to include in subsequent random forest and GAM models.

**Data Availability:** Data are available at Figshare: <https://doi.org/10.6084/m9.figshare.30904115>

**References:**

Fofonoff, P., & Lee, V. (1992). *Appalachian Mountain Club--Rhode Island Salt Pond Watchers Data Report: Water Quality In Trustom And Card’s Ponds 1992*. https://repository.library.noaa.gov

Nixon, S. W., Fulweiler, R. W., Buckley, B. A., Granger, S. L., Nowicki, B. L., & Henry, K. M. (2009). The impact of changing climate on phenology, productivity, and benthic-pelagic coupling in Narragansett Bay. *Estuarine Coastal and Shelf Science*, *82*, 1–18. https://doi.org/10.1016/j.ecss.2008.12.016

Rozier, R. (2015). Evaluation of microbiological water quality in Point Judith Pond (Rhode Island, USA): Quantitation of fecal pollution and presence of human pathogenic bacteria. *Open Journal of Water Pollution and Treatment*, *2015*, 25–32. https://doi.org/10.15764/WPT.2015.01003

Russell, F. D. (2024). *Reconstruction of Late Holocene Relative Sea-Level Changes for the Coastal Ponds of Rhode Island, USA* [M.S., University of Rhode Island]. https://www.proquest.com/docview/3103753363/abstract/88B29884F8044DC6PQ/1
